# Imaging Spectrum of Severe Urinary Tract Infection Complications Presenting as a Seminal Vesicle Abscess, Prostatic Abscess, and Renal Abscess in Uncontrolled Diabetes Mellitus: A Case Report

**DOI:** 10.7759/cureus.48123

**Published:** 2023-11-01

**Authors:** Huma Khan, Suresh Phatak, Kajal Mitra, Sandip Dhote, Wajid Attar

**Affiliations:** 1 Radiology, NKP Salve Institute of Medical Sciences and Research Centre and Lata Mangeshkar Hospital, Nagpur, IND; 2 Radiodiagnosis, Datta Meghe Institute of Medical Sciences, Wardha, IND; 3 Radiodiagnosis and Imaging, NKP Salve Institute of Medical Sciences and Research Centre and Lata Mangeshkar Hospital, Nagpur, IND; 4 Radiodiagnosis, NKP Salve Institute of Medical Sciences and Research Centre and Lata Mangeshkar Hospital, Nagpur, IND

**Keywords:** ultrasonography, magnetic resonance imaging, ct scan, abscess, urinary tract infection complication

## Abstract

Urinary tract infections are more common and severe, and they carry worse outcomes for patients with type 2 diabetes mellitus. The infections are typically caused by resistant pathogens, leading to many complications. Various impairments in the immune system, poor metabolic control, and incomplete bladder emptying due to autonomic neuropathy may all contribute to the enhanced risk of urinary tract infections in these patients.

We present an imaging spectrum of a severe urinary tract infection presenting as renal, prostatic, and seminal vesicle abscesses in a patient with uncontrolled diabetes mellitus.

## Introduction

Diabetes mellitus patients typically have impaired immune systems caused by poor metabolic control and incomplete emptying of the urinary bladder due to autonomic neuropathy, making the patients vulnerable to urinary tract infections. Other risk factors include chronic medical conditions, liver cirrhosis, undergoing hemodialysis for end-stage renal disease, transplants, undergoing chemotherapy, HIV/AIDS, benign prostatic hyperplasia, and immunodeficiency [1,2]. Seminal vesicles are accessory sex organ glands present in the male genitourinary tract and have a key role in male fertility. Various pathologies seen in seminal vesicles are cysts, congenital abnormalities, and infections. An abscess is caused by an obstruction [3]. As the prostate is anatomically close to the seminal vesicles, infection can spread to the prostate and lead to the development of a prostatic abscess. Pathology shows pus and debris within the abscess cavity [4].

## Case presentation

Clinical presentation

A 60-year-old male patient presented with a two-week history of fever, urethral discharge, urinary frequency, hesitancy, poor stream, nocturia, and straining. Rectal examination revealed a smooth tender prostate gland. Investigations on admission showed elevated inflammatory markers. The patient’s medical history included type 2 diabetes mellitus (HbA1c: 11.8%; normal: 4%-5.6%), and the blood sugar level was 300 mg/dl post meal (normal: 140-180 mg/dL).

Diagnostic assessment

The complete blood workup revealed a hemoglobin of 6.9 gm/dL (normal: 12.4-14.9gm/dl), total leukocyte counts of 16900/microliter (normal: 4,500-11,000 WBCs per microliter), and platelet count of 3,27,000 per microliter (normal: 150,000-450,000 platelets per microliter of blood), and serum potassium was 3.7mEq/L (normal: 3.5-5.5 mEq/L) at admission. He is a diabetic male and is on an oral hypoglycemic agent namely SGLT2 inhibitor (dapagliflozin) with negligent control. Urine routine microscopic examination revealed moderately increased albumin, severely increased sugar levels, plenty of pus cells (10-12 per high-power field [HPF]), and RBC of 4-6/HPF (normal range of RBC being <3/HPF). Bacteriuria was also present. The kidney function test revealed creatinine of 3.0 mg/dl (normal: 0.6-1.1 mg/dl) and urea of 30 mg/dl (5-20 mg/dl). The urinary acid-fast bacilli (AFB) test was negative, so genitourinary tuberculosis was ruled out. The patient was treated with broad-spectrum antibiotics including Levofloxacin and Amoxicillin for three weeks, and his medical condition improved.

Response to the treatment was evaluated throughout medical therapy. Clinical symptoms and laboratory results were followed including flank pain, temperature, white blood cell counts, CRP, and ESR. The symptoms and laboratory results all were improved with successful treatment.

**Figure 1:**
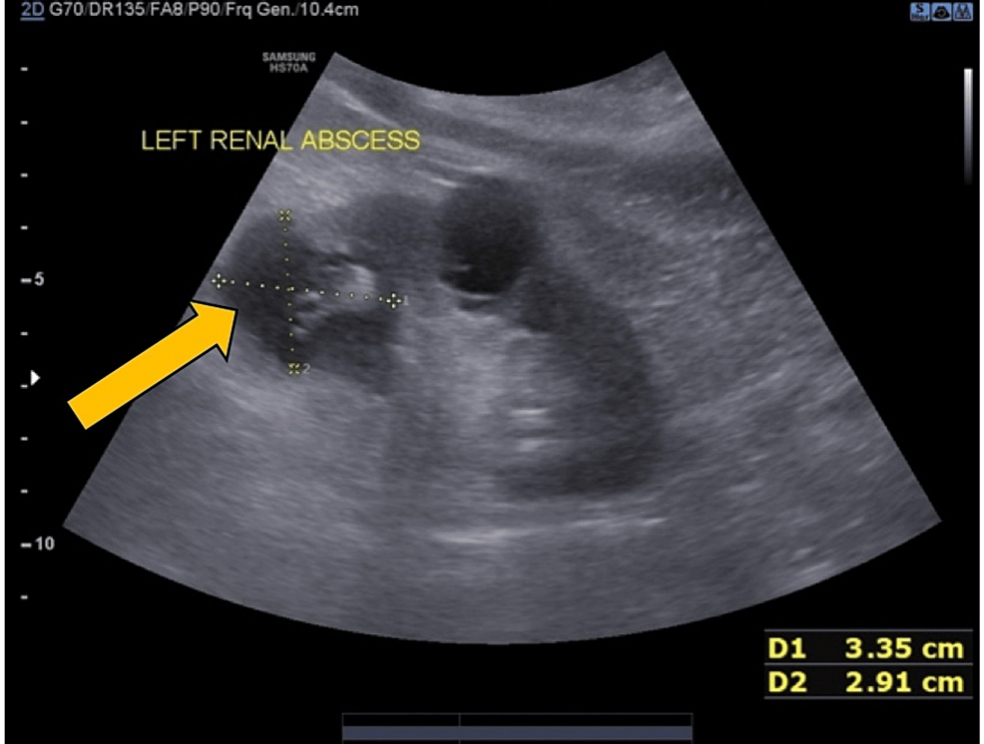
Ultrasound of the abdomen revealing a well-defined hypoechoic cystic lesion with a thin wall with debris and multiple echoes noted at the upper pole of the right kidney

Ultrasound of the abdomen revealed a well-defined hypoechoic cystic lesion with a thin wall with debris and multiple echoes noted at the upper pole of the right kidney. A cortical cyst was noted at the interpolar region (Figure 1).

**Figure 2:**
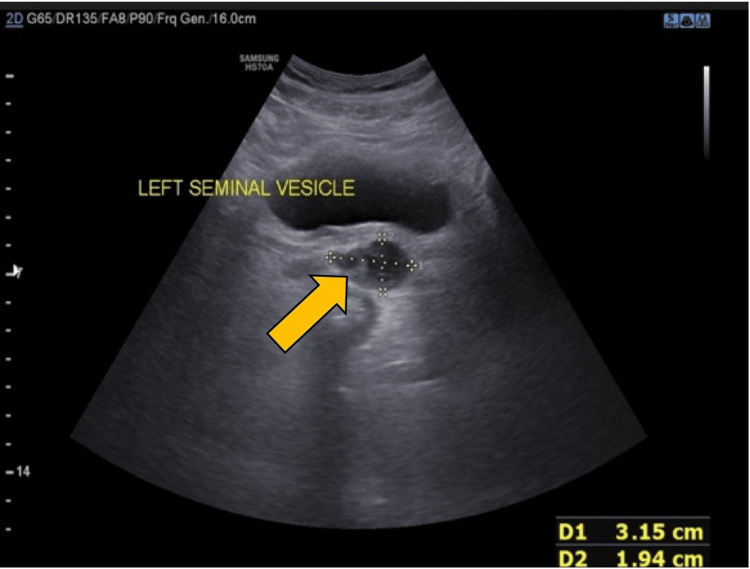
Ultrasonography showing a cystic lesion with multiple echoes in the left seminal vesicle, suggestive of seminal vesicle abscess

On ultrasonography, a cystic lesion with multiple echoes was noted in the left seminal vesicle, suggestive of seminal vesicle abscess (Figure 2).

**Figure 3:**
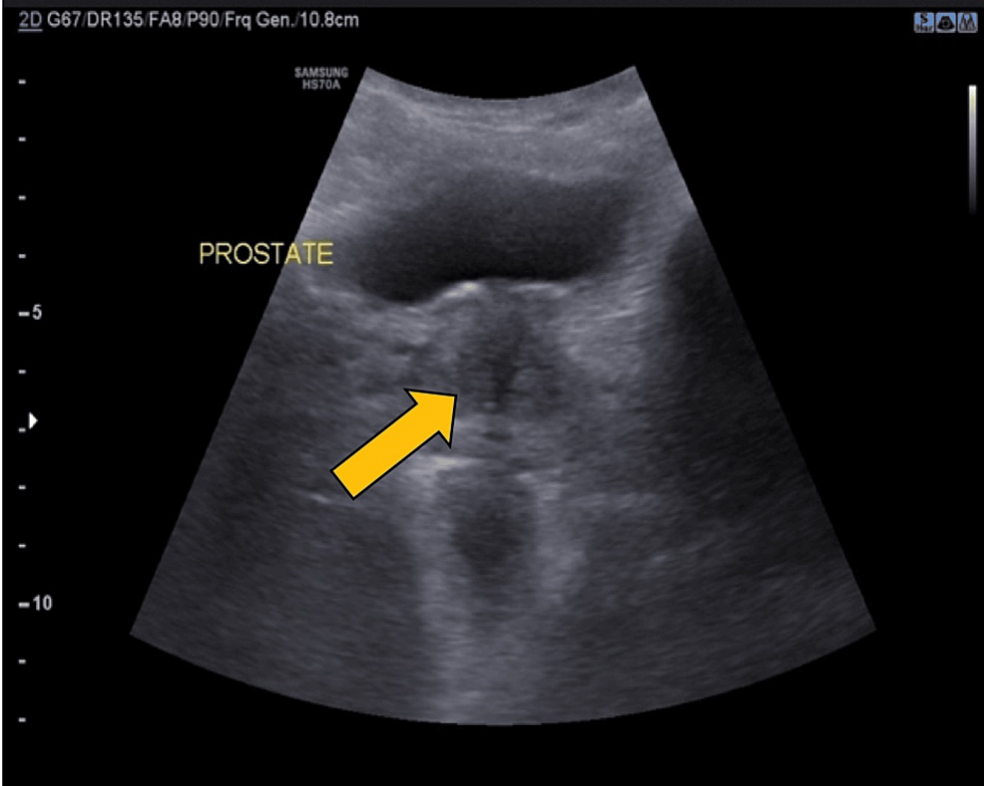
The prostate appearing heterogenous on ultrasonography, suggesting prostatitis

On ultrasonography, the prostate appeared heterogeneous, suggestive of prostatitis (Figure 3).

**Figure 4:**
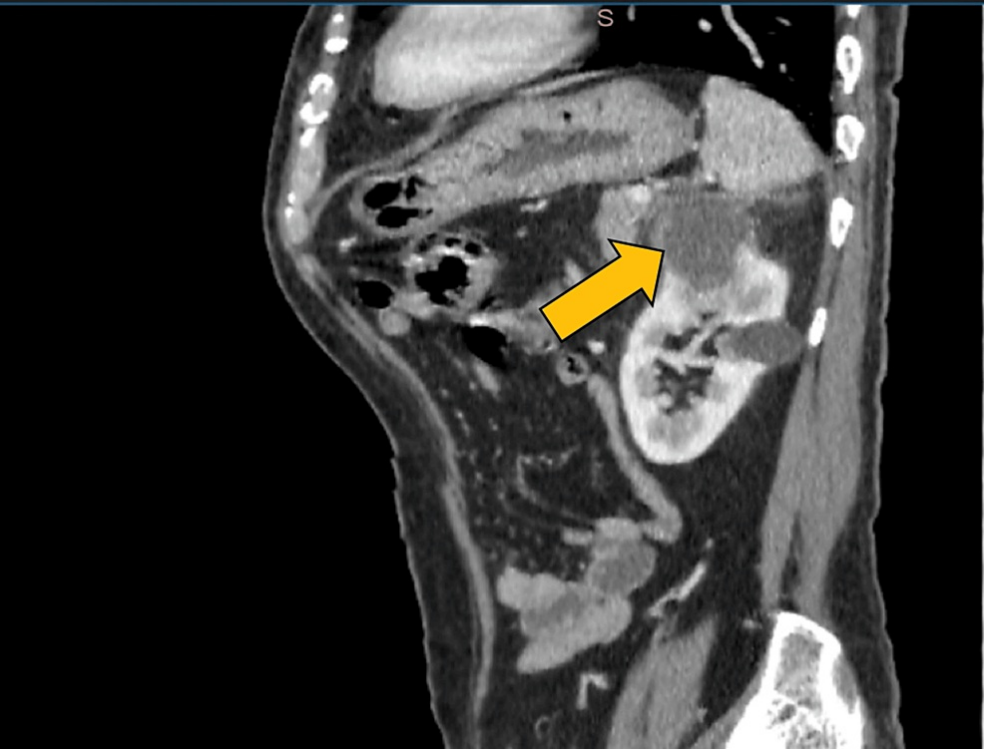
Sagittal CECT showing a relatively well-defined mass of low attenuation with a thick irregular enhancing wall or pseudocapsule at the upper pole of the left kidney CECT: Contrast-enhanced computed tomography.

On sagittal contrast-enhanced computed tomography (CECT), a relatively well-defined mass of low attenuation with a thick irregular enhancing wall or pseudocapsule was noted at the upper pole of the left kidney. Mild perirenal fat stranding was noted, suggestive of renal abscess. A cortical cyst was noted at the interpolar region (Figure 4).

**Figure 5:**
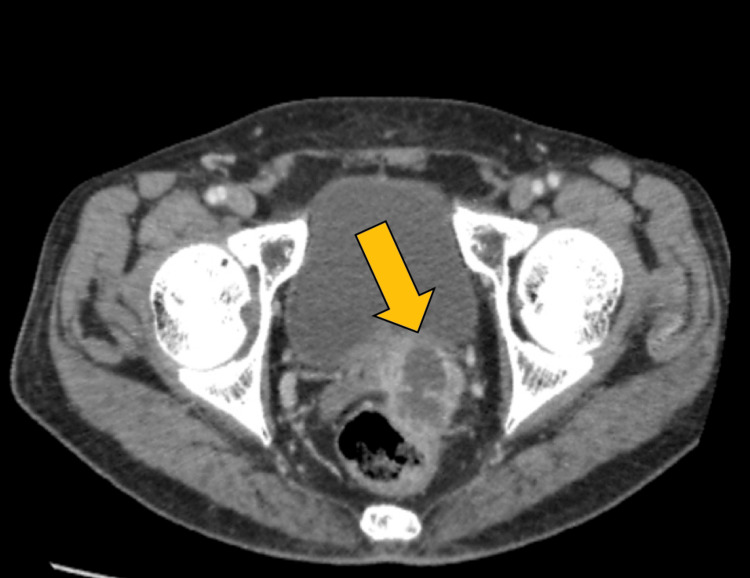
Axial CECT scan showing the cyst with internal echoes in the left seminal vesicle CECT: Contrast-enhanced computed tomography.

An axial CECT scan shows that a cyst with internal echoes was seen in the left seminal vesicle, suggestive of a seminal vesicle abscess (Figure 5).

**Figure 6:**
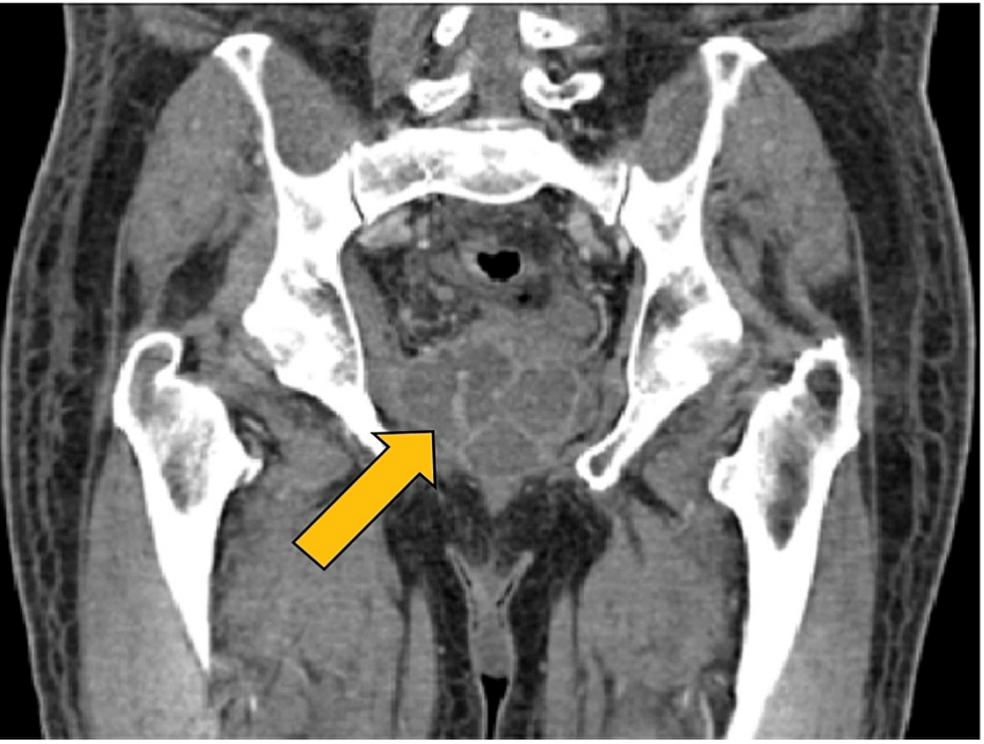
Coronal CECT showing multiloculated hypoechoic lesions with enhancing walls in the prostate CECT: Contrast-enhanced computed tomography.

On coronal CECT, multiloculated hypoechoic lesions with enhancing walls in the prostate were noted, suggestive of a prostatic abscess (Figure 6).

**Figure 7:**
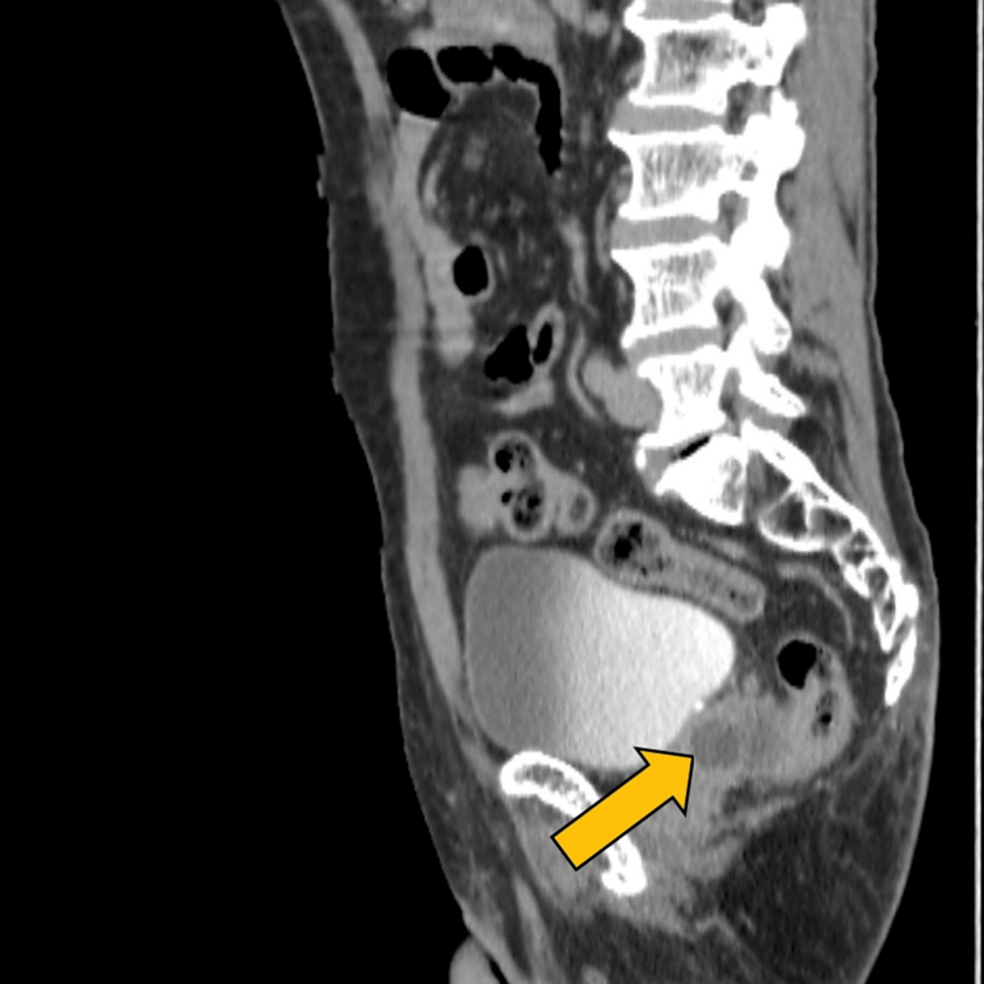
Midsaggital image showing multiloculated hypoechoic lesions with enhancing walls in the prostate CECT: Contrast-enhanced computed tomography.

On a midsagittal image, multiloculated hypoechoic lesions with enhancing walls in the prostate were noted, suggestive of a prostatic abscess (Figure 7).

## Discussion

The pathophysiology of urinary tract abscesses is related to the reflux of organisms into the prostatic ducts, causing acute prostatitis, and the patient’s immunodeficient condition leads to prostatic abscess as a complication [5]. The seminal vesicles neighbor the bladder base, rectum, prostate, ureters, and peritoneum, which can present the symptoms of seminal vesicle inflammation. Abscesses like fever, urethral discharge, urinary frequency, and hesitancy are often overlapping and variable [6].

Commonly encountered organisms are gram-negative *Escherichia coli*, followed by *Klebsiella*, *Enterobacter*, *Proteus*, *Pseudomonas*, *Serratia*, and *Enterococcus *species as well as *Staphylococcus aureus* [7].

A seminal vesicle abscess is best imaged on contrast-enhanced CT, showing an enlarged seminal vesicle associated with hypodense areas within, fat stranding in adjacent areas, and occasionally a thickened urinary bladder wall [8,9]. On MRI, inflammation of the seminal vesicle is seen as a hypointense lesion on T1-weighted images and hyperintensity on T2-weighted images. MRI is valuable in showing the invasion of abscesses to adjacent organs like the prostate [10].

Radiographic findings of a renal abscess may include the absence of a psoas muscle margin associated with an enlarged kidney, ill-defined renal margins, and radio opacity in the renal fossa. On the ultrasound, debris and fluid levels are visualized [11]. On CT, a renal abscess is seen as a non-enhancing area with decreased attenuation and enhancement of walls. Perinephric spread of renal abscess is best visualized on contrast-enhanced CT [12].

Transrectal ultrasound (TRUS) is the modality of choice for diagnosing a prostate abscess seen as a hypoechoic lesion with septations [13]. Multidetector computed tomography (MDCT) features of a prostatic abscess are enlargement and multiple fluid collection with peripheral enhancement. Advantages of MDCT include extraprostatic spread into paravesical space, perineum, ischiorectal fossa, and rectum. CT is also useful in monitoring the treatment [14]. On MRI, abscesses are seen as hypointense on T1-weighted images and hyperintense on T2-weighted images, which is heterogeneous and has thick walls. On diffusion-weighted images, restricted diffusion is seen [15]. On post-contrast imaging, thick-walled fluid collection is visualized and helps detect the extraprostatic spread of abscess [16].

## Conclusions

Urinary tract infections in uncontrolled diabetic patients result in serious complications. Due to advancements in cross-sectional imaging techniques, lesions of the seminal vesicle, prostate, and kidney can be diagnosed with high accuracy. Ultrasonography and CT scans are the imaging modalities of choice in diagnosing abscesses in the urinary tract, helping early diagnosis and patient management. Antibiotics are the initial course of treatment. Abscesses should only be transperineally, transurethrally, or percutaneously drained in situations where antibiotic therapy has failed. Surgical treatment is the final option for situations in which the previous procedures failed.
